# Fatal Outcome of Membranoproliferative Glomerulonephritis in a Patient With Hidden Visceral Leishmaniasis

**DOI:** 10.1155/crdi/3448351

**Published:** 2025-05-19

**Authors:** Ali Mansoursamaei, Maryam Valikhani, Hoofar Rafiee

**Affiliations:** ^1^School of Medicine, Shahroud University of Medical Sciences, Shahroud, Iran; ^2^Clinical Research Development Unit, Imam Hossein Hospital, Shahroud University of Medical Sciences, Shahroud, Iran

**Keywords:** glomerulonephritis, leishmaniasis, membranoproliferative, visceral

## Abstract

**Background:** Visceral leishmaniasis (VL) is a systemic parasitic disease with diverse clinical manifestations, primarily affecting the spleen, liver, and bone marrow. While renal involvement in VL is well documented, it is often mild and resolves with effective treatment. However, severe renal complications, such as membranoproliferative glomerulonephritis (MPGN), are rare and typically associated with immunocompromised individuals.

**Case Report:** We report the case of a 30-year-old male from a nonendemic region who presented with unusual clinical features of generalized edema and abdominal pain, without the classic signs of VL such as fever or hepatosplenomegaly. Clinical examination, laboratory investigations, and other therapeutic interventions ruled out hepatic and cardiac causes of edema, focusing the diagnosis on nephrotic syndrome and acute kidney injury (AKI). A renal biopsy revealed MPGN, a rare but documented complication of VL. The presence of an autoimmune disease was suspected initially, and the patient was treated with steroids without improvement. The diagnosis of VL was confirmed only after bone marrow biopsy, which identified Leishmania amastigotes. Despite prompt initiation of antileishmanial therapy with liposomal amphotericin B, the patient's renal function continued to diminish, which finally resulted in end-stage renal disease (ESRD). The patient later developed pleural and pericardial effusion, pulmonary embolism, and tamponade, ultimately leading to a fatal outcome.

**Conclusion:** This case underscores the importance of considering VL in the differential diagnosis of unexplained renal dysfunction, even in the absence of classic symptoms and in nonendemic regions. It highlights the potential for VL to present with atypical renal manifestations such as MPGN, leading to severe and irreversible renal damage if diagnosis and treatment are delayed. Early recognition and intervention are critical to improving outcomes in VL-associated renal disease.

## 1. Introduction

Leishmaniasis is a neglected infectious disease caused by protozoan parasites and transmitted by the bite of certain infected species of sand flies (subfamily Phlebotominae). More than 20 species of Leishmania are responsible for the four major clinical syndromes in humans, including cutaneous leishmaniasis, mucocutaneous leishmaniasis, visceral leishmaniasis (VL), and Kala-Azar dermal leishmaniasis. [1–3]. VL, primarily caused by *Leishmania donovani* and Leishmania infantum, is a systemic disease that accounts for approximately 500,000 new cases and 50,000 deaths globally yearly [4]. This systemic disease caused by L. infantum is common in several Mediterranean countries such as Iran. The southern and northwestern regions of Iran are known as endemic regions for VL with 100–300 cases per year [5]. VL diagnosis traditionally relies on the direct visualization of Leishmania parasites through microscopy or culture from invasive samples such as the spleen, bone marrow, or lymph node aspirates. Serological tests, particularly the rK39 rapid diagnostic test (RDT), ELISA, and the direct agglutination test (DAT), alongside the molecular diagnostics, like PCR have become crucial, offering high sensitivity [6].

The clinical manifestations of VL are diverse depending on the Leishmania species, genetic factors, and immunological characteristics of the patients. After an incubation period, classic clinical symptoms include fever, fatigue, and weight loss. As the disease progresses, hepatosplenomegaly, anemia, pancytopenia, hypergammaglobulinemia, and elevated autoantibodies may be observed in these patients [1–3, 7]. Autoimmune manifestations such as cutaneous vasculitis, high titers of rheumatoid factor (RF), serum cryoglobulins, and complement consumption are frequently observed. Subsequent symptoms include cachexia, liver dysfunction with jaundice, hypoalbuminemia, and edema [1, 3, 8]. These manifestations might persist for weeks to months and, in case of lack or delay in treatment, patients may die from bacterial coinfections, massive bleeding, or severe anemia [9]. There is therefore a large heterogeneity of clinical manifestations, and sporadically renal involvement can also be observed.

Several studies have highlighted various types of renal manifestations in patients with VL, further underscoring the complexity of VL nephropathy. For instance, in the cohort study of 50 patients with VL by Dutra et al. [10], proteinuria and/or microscopic hematuria or pyuria were observed in 51% of patients. There was a tubulointerstitial involvement in all seven patients who underwent kidney biopsy with a proliferative glomerulonephritis in five of them. Also, Daher et al. [11] noted the occurrence of acute kidney injury (AKI) in 26.3% of 57 VL patients. Individual case reports have also provided insights into rare but severe renal complications, such as the granulomatous pyelonephritis described by Romero Maroto et al. [12], the segmental necrotizing glomerulonephritis with 70% crescents on kidney biopsy reported by Chaigne et al. [13], and subacute renal failure, which caused by membranoproliferative glomerulonephritis (MPGN) by Romero et al. [8]. These reports collectively underscore the broad spectrum of renal involvement in VL, ranging from mild proteinuria to severe glomerulonephritis and AKI, necessitating a broad approach to diagnosis and management.

In this study, we present a rare case of leishmaniasis infection with unusual clinical manifestations in a nonendemic region for leishmaniasis.

## 2. Case Report

A 30-year-old man was admitted to the emergency department in June 2020 for abdominal pain extending to the testes and generalized edema. The patient did not mention fever, anorexia, cough, dyspnea or orthopnea, weight loss, nausea or vomiting, hematuria, and oliguria. He had a history of kidney stones and lithotripsy, opioid dependence (since 2015), and was a smoker (10 p/y). However, he had no history of any other disease, medication, or alcohol consumption. On travel history, the patient had a very short trip (1 week) to Sistan and Baluchestan province, an endemic leishmaniasis zone [5]. On clinical examination, the patient appeared ill. His blood pressure was 130/80 mmhg, pulse rate 90, respiratory rate 19, and temperature 37.2°C. There was no sign of pallor, jaundice, cyanosis, skin rashes, joint swelling, redness or pain, and muscle pain. Mental status, neurologic examinations, and chest auscultation were normal. On inspection, the abdomen was normal, and on palpation, it was soft. Also, tenderness or rebound tenderness was not presented on examination. A 2+ pitting edema in the upper extremities, 4+ pitting edema in the lower extremities, and periorbital edema were detected. However, there was no evidence of Jugular venous distension, S3 heart sound, hepatomegaly, or ascites which was proponent of the renal origin of edema. Further examinations revealed no sign of photosensitivity, alopecia, oral ulcers, Raynaud's phenomenon, morning stiffness, joint deformities or nodules, dry mouth or eye, goiter, constipation, nail changes, and blood in stool on digital rectal examination. Laboratory examinations at the time of the first referral and after treatment are summarized in Table 1. ECG and eco-cardiogram were performed and found to be normal; troponin level was checked twice and was in normal range (< 9). The results of vein blood gas revealed Ph 7.32, hco3 21 mmol/L, and pco2 45 mmhg. Blood and urine culture were run and were both negative. Due to the high ESR and CRP levels, a COVID-19 PCR test and chest radiograph were performed and found to be normal. Due to his drug addiction and suspected HIV infection, he was tested for HIV twice, and at both times, the test was negative. HBs antigen and HCV antibodies were measured by ELISA and were negative. Also, immunoglobulin M and G (IgM and IgG) for Epstein–Barr virus (EBV) and cytomegalovirus (CMV) were checked and both are negative. Abdominopelvic sonography and renal Doppler ultrasound were performed and reported normal. So, he was hospitalized with a diagnosis of nephrotic syndrome and AKI. The patient then underwent dialysis due to the elevated creatinine and generalized edema that did not respond to initial medical treatment.

**Figure 1:**
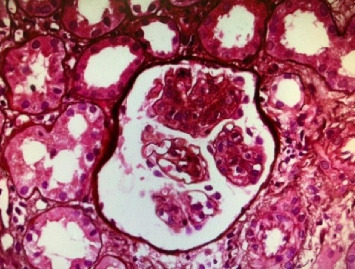
Lobular accentuation with endocapillary and mesangial proliferation and GBM thickening. H&E ×100.

A renal biopsy was performed to confirm the diagnosis. In the results of the light microscope, lobular accentuation with endocapillary and mesangial proliferation and GBM thickening was seen with no significant vasculopathy or amyloid deposition. Also, 20% of the tubules were atrophic, and few tubules contained hyaline and RBC cast. Immunofluorescence microscopy revealed granular deposits along the GBM and mesangial of C3c (2+), C1q (1+), ig G (2+), ig A (1+), kappa (1+), and lambda (1+); Ig M and fibrinogen were negative. Thus, histologic examination of the renal tissue showed MPGN in a capillary lumen. The result of the renal biopsy revealed MPGN (Figure 1). A course of 30 mg/day oral prednisolone was started and continued for 1 month because rheumatic disease including systemic lupus erythematosus was suspected, followed by a tapering regimen. Secondary causes were also investigated during the examination.

**Figure 2:**
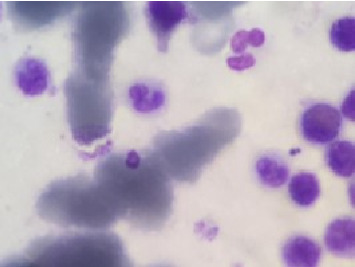
Leishmania parasites are observed in a bone marrow aspirate. Original magnificent 100x.

Due to a suspicion of rheumatological diseases, the concentrations of several indicators were measured. The antinuclear antibody (ANA) was measured at 7 U/mL, perinuclear antineutrophil cytoplasmic autoantibodies (P-ANCA) were at 10 U/mL, antineutrophil cytoplasmic antibodies (C-ANCA) were at 9 U/mL, RF was at 8 U/mL, anti–double-stranded DNA antibodies (Anti-ds-DNA) were 22 U/mL, and the complement Components 3 and 4 (C3 and C4) were 90 and 10 U/mL, respectively. All of these values were found to be within the normal range. During hospitalization, his creatinine increased and he was found to have severe unwarrantable anemia with a hemoglobin level of 6. Because of the unjustifiable anemia and to rule out other systemic disease, a bone marrow biopsy was performed. In bone marrow aspiration, megakaryocytes with amastigotes of Leishmania were seen under the microscope, confirming the diagnosis of VL (Figure 2) (Figure 2). With the diagnosis of VL, administration of 3 mg per kg of intravenous liposomal amphotericin B was started immediately on Days 1 to 5, 14, and 21. After leishmaniasis diagnosis, prednisolone was discontinued. Due to the unavailability of the PCR or ELISA, a second bone marrow aspiration was performed after the completion of the treatment period to check VL response to the treatment, and no sign of the disease was observed. Despite treatment, the patient's renal function did not improve and creatinine remained elevated, and the patient eventually developed end-stage renal disease (ESRD). Therefore, the patient was dialysis-dependent and referred for dialysis three times per week for 4 months. Due to the lack of improvement in kidney function, a second renal biopsy should have been performed to further evaluate the ongoing proliferative glumerulonephritis. However, due to the low socioeconomic conditions and financial insufficiency, the patient did not consent to the second biopsy. In November 2020, he again presented to the emergency department with chest pain, shortness of breath, and low blood pressure. Results of echocardiography showed moderate right ventricle (RV) and left ventricle (LV) dysfunction with 30% ejection fraction. Global hypokinesis and septal akinesis were also reported. Chest X-ray revealed a massive pleural effusion, cardiomegaly, and tamponade, so pericardiocentesis and massive dialysis were performed urgently. Measured pleural effusion volume by sonography in left and right side were 120 and 1000 cc, respectively. After 1 week of hospitalization, a filling defect in the inferior lobe of the left lung was observed by lung CT-angiography, which was originator of pulmonary emboli. Patient's clinical condition deteriorated over a week, and the patient expired from the pulmonary embolism and tamponade.

## 3. Discussion

Renal involvement can be an important feature of VL that is usually mild and limited with effective infection control and might be associated with increased morbidity and mortality. There are limited studies of renal disease caused by VL. In general, leishmaniasis-associated renal disease occurs more in immunocompromised patients [8, 14]. Renal involvement can progress from proteinuria, hematuria, and leukocytopenia to AKI and ESRD [8]. Data on renal prognosis are limited, but it has been reported that AKI may contribute to poor patient outcomes [3]. Our patient was referred to the hospital with AKI initially. AKI in nephrotic syndrome can be precipitated by a variety of factors. There was no evidence of hemodynamic instability, such as hypotension or tachycardia that would suggest prerenal AKI. Moreover, absence of severe dehydration and a lack of nephrotoxic drug exposure pointed away from ATN as the primary cause. Urine sediment was not suggestive of ATN, and the clinical picture did not indicate sepsis or shock-related renal injury. RPGN was also suspected; however, the lack of serological markers for autoimmune diseases (negative ANA and RF) and specific renal biopsy findings (absence of crescents) made this diagnosis unlikely.

Leishmaniasis can cause tubular and glomerular dysfunction, especially in chronic forms. The most frequent histological finding in these patients is interstitial nephritis, but MPGN has rarely been reported [15, 16]. MPGN is a pattern of glomerular injury characterized by mesangial hypercellularity, endocapillary proliferation, and capillary wall remodeling with double contour formation [17]. Based on immunofluorescence findings, MPGN is now categorized as either immune complex-mediated or complement-mediated. Immune complex-mediated MPGN can be associated with various conditions, including chronic infections (such as hepatitis C and endocarditis), autoimmune diseases (such as lupus), and monoclonal gammopathies, where circulating immune complexes are deposited in the kidneys [18]. Renal biopsy can further differentiate these causes by showing specific patterns of immune deposits, helping tailor the management and treatment approach. The association between VL and MPGN is well-documented, although relatively rare [3]. In our case, the temporal relationship between VL diagnosis and MPGN, along with the absence of other common causes, strongly suggests a causal link. Also, the presence of both immunoglobulins and complement components suggests an ongoing immune response to persistent antigens, which is characteristic of infection-related MPGN, highlighting the importance of considering infectious causes in the differential diagnosis of MPGN, especially in endemic areas or in patients with relevant travel history [19, 20].

MPGN caused by infections is usually associated with immune complexes and characterized by the presence of immunoglobulins (usually IgG and IgM) and complement factors [3, 14, 21]. The infection stimulates T helper 2 lymphocytes, which produce interleukins (IL)-4 and IL-10. These interleukins activate B-cells to produce a wide range of antibodies, leading to glomerular damage triggered by the chronic parasitic infection [22]. Although immune complex glomerulonephritis is rare, a high prevalence of autoantibodies and circulating immune complexes is a common finding in chronic VL [1]. Contrary to the claim, our patient had immune complex glomerulonephritis and serum electrophoresis was normal. The diagnosis of VL was made following the discovery of Leishmania bodies in the bone marrow biopsy. This probably indicated an infectious cause of MPGN, with glomerular damage due to the immune complex deposition.

Notably, persistence of renal dysfunction despite treatment of the underlying infection, as observed in our patient, underscores the complexity of MPGN pathogenesis and the potential for irreversible glomerular damage in cases of delayed diagnosis and treatment [7]. Hemodynamics is disturbed in the context of the disease caused by hypovolemia, hypoalbuminemia, or anemia, and the different drugs focused on therapy (i.e., amphotericin B) can also be involved in renal damage [1, 3, 14]. Some of the treatment methods of VL such as amphotericin B and pentamidine may also cause renal toxicity. While many cases of VL-associated renal disease improve with antileishmanial treatment, persistence or progression of renal dysfunction despite successful VL therapy is not uncommon [3]. However, despite the control of VL, renal dysfunction did not return to normal in our case. The patient had anemia (Hb = 6 g/dL) and hypoalbuminemia (Alb = 2.1 g/dL); thus, the deposition of immunoglobulins and complement factors, anemia, and hypoalbuminemia accelerated ESRD.

Leishmaniasis can be easily misdiagnosed with connective tissue diseases and rheumatic diseases such as rheumatoid arthritis (RA), systematic lupus erythematosus (SLE), scleroderma, or even immune thrombocytopenia (ITP) especially in immunocompromised patients such as HIV-infected patients [1]. If treatment is incomplete or the disease is misdiagnosed, VL patients may die from bacterial coinfection, hemorrhage, or severe anemia [3]. On admission to the hospital, our patient had only edema and proteinuria, and there were no signs of systemic disease such as fever, pancytopenia, hepatosplenomegaly, or hypergammaglobulinemia. Therefore, the diagnosis was made 2 months after the first sign of the disease, which was edema. And because of the young age of the patient and the nephrotic syndrome, rheumatic and renal diseases were more plausible. Also, despite the MPGN diagnosis, there was no evidence of leishmaniasis on renal biopsy. In fact, the final diagnosis was made incidentally after the bone marrow biopsy because of a sharp decrease in hemoglobin (Hb = 6). The pathogenesis of MPGN in VL is primarily due to immune complex deposition rather than direct parasite invasion of the kidney. So, the absence of visible parasites does not rule out VL as the underlying cause of MPGN, as seen in other studies [8, 23]. In addition, a large number of differential diagnoses prolonged the diagnostic process. In this regard, Romero et al. featured a patient presenting with renal failure due to MPGN without typical VL symptoms such as hepatosplenomegaly [8]. Similarly, autoimmune diseases were initially suspected, leading to ineffective steroid treatment, and also, the correct diagnosis of VL was only reached after extensive investigations, including renal and bone marrow biopsies, highlighting the potential for VL to mimic autoimmune diseases, emphasizing the need for a comprehensive diagnostic approach in complex presentations of renal dysfunction. Therefore, early diagnosis is critical, especially in endemic regions. A range of diagnostic methods, including PCR and serological tests such as ELISA and the rk39 test, are used to detect VL. The rk39 test is a widely used, noninvasive serological test for diagnosing VL [24]. It is particularly effective in endemic areas, providing a quick and efficient diagnostic tool [25]. Moreover, PCR-based methods provide superior accuracy and are especially valuable in HIV coinfection cases or when serological tests yield inconclusive results [26].

Finally, the patient's clinical condition worsened, although secondary bone marrow aspiration did not reveal leishmaniasis. If the diagnosis had been made earlier, the therapeutic approach would have been different, and this could have helped us to make the diagnosis more quickly, preserve the function of the kidney, or perhaps prevent mortality. The patient's deterioration culminated in a fatal outcome due to complications arising from ESRD, including severe pericardial and pleural effusion that led to cardiac tamponade. These complications are common in ESRD due to volume overload, uremic state, and impaired kidney function [27]. Additionally, the patient experienced pulmonary embolism due to hypercoagulability associated with renal dysfunction, which contributed to the extra strain on the cardiovascular system. Notably, the patient lived in Semnan province, a nonendemic region for leishmaniasis, but he had a very short trip (1 week) to Sistan and Baluchestan province, an endemic leishmaniasis zone [5, 28]. Therefore, travel to epidemic and endemic regions should be considered when leishmaniasis is suspected.

## 4. Conclusion

This case report brings attention to several significant aspects. Firstly, it highlights the rare occurrence of MPGN and immune complex glomerulonephritis as potential renal complications of VL, particularly noteworthy in endemic and epidemic regions. While previous VL cases associated with MPGN predominantly involved immunocompromised individuals, our patient, despite being young and healthy with no underlying systemic diseases, presented with this complication. Secondly, despite receiving complete treatment for leishmaniasis, the patient's renal function deteriorated, ultimately leading to ESRD, likely due to delayed diagnosis. This underscores the critical importance of early recognition and prompt intervention in VL cases to prevent severe complications and preserve renal function.

## Figures and Tables

**Table 1:** Laboratory investigations.

	Initial values	After Rx	Reference range
Hb (gr/dL)	10.5	8.6	14–16
WBC (×10^3^/mL^3^)	8.9	9.9	4–10
Neutrophil (%)	86%	88%	< 75%
Plt (×10^3^/mL^3^)	284	296	150–450
AST (U/L)	35	68	< 37
ALT (U/L)	16	11	< 41
ALK (U/L)	324	304	80–306
Bilirubin (total/direct) (mg/dL)	0.5/0.4	0.5/0.2	0.3–1.2/< 0.3
BUN (mg/dL)	54	68	15–45
Cr (mg/dL)	2.5	4.1	0.9–1.3
Na (mEq/L)	133	124	136–145
K (mEq/L)	5.6	5.5	3.5–5.5
Ca (mg/dL)	8.6	8.3	8.5–10.2
Albumin (mg/dL)	2.1	3.1	3.4–5.4
ESR (mm)	90	110	< 15
CRP (qualitative)	3+	2+	Negative
Amylase (U/L)	71	38	30–100
Lipase (U/L)	22	25	10–60
*Urine analysis*			
Color	Yellow	Yellow	Yellow
Clarity	Semiturbid	Semiturbid	Clear
PH	5.5	5	5.5–7.5
Specific gravity	1.020	1.030	1.005–1.030
Protein	4+	2+	Negative to trace
Ketones	Negative	Negative	Negative
Leukocyte esterase	Negative	Negative	Negative
Nitrite	Negative	Negative	Negative
Glucose	Negative	Negative	Negative
WBC (cells per high-power field)	8–10	12–14	0–5
RBC (cells per high-power field)	0	1–2	0–2
Bacteria (per high-power field)	Few	Few	None to few
Casts (per high-power field)	0	0	0–3
Crystals	Few amorph urate	Few amorph urate	None to few
24 h protein in urine (per day)	4600 mg	2200 mg	< 150 mg

*Note:* Hb, hemoglobin; Plt, platelet; Cr, creatinine; Na, sodium; K, potassium; Ca, calcium.

Abbreviations: ALP, alkaline phosphatase; ALT, alanine aminotransferase; AST, aspartate transaminase; BUN, blood urea nitrogen; CRP, C-reactive protein; ESR, erythrocyte sedimentation rate; WBC, white blood cell.

## Data Availability

Data sharing is not applicable to this article as no new data were created or analyzed in this study.
